# EXPLORING TECHNOLOGY-ENABLED ASSESSMENTS OF SYMPTOMS EXPERIENCE BY INFORMAL CAREGIVERS

**DOI:** 10.1093/geroni/igz038.1220

**Published:** 2019-11-08

**Authors:** Daniel J Schulman, Portia Singh, Mladen Milosevic, Ali Samadani

**Affiliations:** 1 Philips Research North America, Cambridge, Massachusetts, United States; 2 Philips Research, Cambridge, Massachusetts, United States

## Abstract

For community-dwelling older adults with chronic conditions, effective symptom management is a determinant of quality of life. Providers often have poor knowledge of an individual’s symptoms experience, especially when contact is infrequent, leading to suboptimal symptom management. Many older adults receive frequent care and contact from family, friends, and other informal caregivers (ICGs). Subjective observation by ICGs is an underexplored information source, but faces barriers including ICG burden and lack of ICG knowledge. It is unclear what relevant information might be collected by ICG observations. We conducted a pilot evaluation of Philips CarePartners Mobile (CPM), a prototype smartphone application that provides communication and coordination support to a “circle” of ICGs assisting an older adult. CPM includes features enabling ICGs to share semi-structured observations. 19 caregivers (in 8 circles) used CPM for 12 weeks, contributing 397 observations and participating in interviews and other assessments. We performed a qualitative analysis of the observations, coding for presence of content relevant to dimensions in the UCSF Symptom Management Theory (perception of, evaluation of, and response to symptoms). Relevant content was found in 150 observations, with perception and assessment more common (141) than response (32). Common symptoms included mobility difficulty (31), fatigue (23), dizziness (21), pain (19), and confusion (18). Among observations without symptom-relevant content, many reported on overall mood (92), and reference to social activities was frequent. These results demonstrate that symptoms experience can be assessed using caregiver observations, although further work may be needed to enable caregivers to provide a comprehensive assessment.

